# *EDA*, *EDAR*, *EDARADD* and *WNT10A* allelic variants in patients with ectodermal derivative impairment in the Spanish population

**DOI:** 10.1186/s13023-019-1251-x

**Published:** 2019-12-03

**Authors:** María Carmen Martínez-Romero, María Juliana Ballesta-Martínez, Vanesa López-González, María José Sánchez-Soler, Ana Teresa Serrano-Antón, María Barreda-Sánchez, Lidya Rodriguez-Peña, María Teresa Martínez-Menchon, José Frías-Iniesta, Paloma Sánchez-Pedreño, Pablo Carbonell-Meseguer, Guillermo Glover-López, Encarna Guillén-Navarro, GIEDE (Spanish multidisciplinary research group for ectodermal dysplasia), Rebeca Alcalá-García, Ana Barcia-Ramírez, Jaime Cruz-Rojo, Blanca Gener-Querol, Angela Hernández-Martín, Pablo Lapunzina-Badía, Isabel Llanos-Rivas, Sabel Lorda-Sánchez, Antonio Martínez-Carrascal, José-Manuel Mascaró-Galy, Lucero Noguera-Morel, María Ángeles Rodríguez-González, Jaime Sánchez del Pozo, Verónica Seidel, Antonio Torrelo, Mª. José Trujillo-Tiebas

**Affiliations:** 1 Centro de Bioquímica y Genética Clínica, Hospital Clínico Universitario Virgen de la Arrixaca, IMIB- Arrixaca. Murcia. CIBERER-ISCIII, Madrid, Spain; 2 Programa de doctorado en Ciencias de la Salud, Universidad Católica de Murcia (UCAM), Murcia, Spain; 3 Sección Genética Médica. Servicio de Pediatría. Hospital Clínico Universitario Virgen de la Arrixaca. IMIB- Arrixaca, Universidad de Murcia. CIBERER-ISCIII, Madrid, Spain; 4 Cátedra de Genética. Facultad de Ciencias de la Salud, Universidad Católica de Murcia (UCAM), Murcia, Spain; 5 Servicio de Dermatología. Hospital Clínico Universitario Virgen de la Arrixaca, Universidad de Murcia, Murcia, Spain; 6 Departamento de Cirugía, Pediatría, Obstetricia y Ginecología. Facultad de Medicina, Universidad de Murcia, Murcia, Spain; 7 Sección Genética Médica (Hospital Materno-Infantil. Planta 0), Hospital Clínico Universitario Virgen de la Arrixaca, Ctra. Madrid-Cartagena s/n, El Palmar, CP 30120 Murcia, Spain

**Keywords:** Ectodermal derivative impairment, hypohidrotic ectodermal dysplasia, Non-syndromic tooth agenesis, Hypodontia, *EDA*, *EDAR*, *EDARADD*, *WNT10A*

## Abstract

**Background:**

Ectodermal dysplasias (ED) are a group of genetic conditions affecting the development and/or homeostasis of two or more ectodermal derivatives. An attenuated phenotype is considered a non-syndromic trait when the patient is affected by only one impaired ectodermal structure, such as in non-syndromic tooth agenesis (NSTA) disorder. Hypohidrotic ectodermal dysplasia (HED) is the most highly represented ED. X-linked hypohidrotic ectodermal dysplasia (XLHED) is the most common subtype, with an incidence of 1/50,000–100,000 males, and is associated with the *EDA* gene (Xq12-q13.1); the dominant and recessive subtypes involve the *EDAR* (2q13) and *EDARADD* (1q42.3) genes, respectively. The *WNT10A* gene (2q35) is associated more frequently with NSTA.

Our goal was to determine the mutational spectrum in a cohort of 72 Spanish patients affected by one or more ectodermal derivative impairments referred to as HED (63/72) or NSTA (9 /72) to establish the prevalence of the allelic variants of the four most frequently associated genes. Sanger sequencing of the *EDA*, *EDAR*, *EDARADD* and *WNT10A* genes and multiplex ligation-dependent probe amplification (MLPA) were performed.

**Results:**

A total of 61 children and 11 adults, comprising 50 males and 22 females, were included. The average ages were 5.4 and 40.2 years for children and adults, respectively. A molecular basis was identified in 51/72 patients, including 47/63 HED patients, for whom *EDA* was the most frequently involved gene, and 4/9 NSTA patients, most of whom had variants of *WNT10A*. Among all the patients, 37/51 had variants of *EDA*, 8/51 had variants of the *WNT10A* gene, 4/51 had variants of *EDAR* and 5/51 had variants of *EDARADD.* In 42/51 of cases, the variants were inherited according to an X-linked pattern (27/42), with the remaining showing an autosomal dominant (10/42) or autosomal recessive (5/42) pattern. Among the NSTA patients, 3/9 carried pathogenic variants of *WNT10A* and 1/9 carried *EDA* variants. A total of 60 variants were detected in 51 patients, 46 of which were different, and out of these 46 variants, 12 were novel.

**Conclusions:**

This is the only molecular study conducted to date in the Spanish population affected by ED. The *EDA*, *EDAR*, *EDARADD* and *WNT10A* genes constitute the molecular basis in 70.8% of patients with a 74.6% yield in HED and 44.4% in NSTA. Twelve novel variants were identified. The *WNT10A* gene has been confirmed as the second molecular candidate that has been identified and accounts for one-half of non-*EDA* patients and one-third of NSTA patients. Further studies using next generation sequencing (NGS) will help to identify other contributory genes in the remaining uncharacterized Spanish patients.

## Background

Ectodermal dysplasias (ED) are a group of genetic conditions affecting the development and/or homeostasis of two or more ectodermal derivatives. An attenuated phenotype has been considered to be a non-syndromic trait when the patient is affected by only one impaired ectoderm-derived structure [1]. ED is a large and heterogeneous group of congenital disorders affecting the normal development of ectoderm-derived structures such as hair, nails, teeth and eccrine sweat glands [2–6]. Hypohidrotic ectodermal dysplasia (HED) (OMIM: # 305100; ORPHA: 238468) is the most common type of ED. HED is due to pathogenic variants in several genes that encode components of the tumour necrosis factor α (TNFα)-related signalling pathway [7]. Pathogenic variants of these genes interfere with the interaction between surface-localized epithelial cells and the underlying mesenchyme, which occurs during embryonic development [8].

It has been demonstrated that HED is caused by pathogenic variants in several genes, including *EDA* (OMIM 300451), which is located on chromosome Xq12-q13.3 and encodes the ligand ectodysplasinA-A1 (EDA-A1), *EDAR* (OMIM 604095), which is located on chromosome 2q11–13 and encodes the ectodysplasinA-A1 receptor, and *EDARADD* (OMIM 606603), which is located on chromosome 1q42-q43 and regulates the structure of EDAR-associated death domain protein. With the exception of the *EDA* gene, which is located on the X chromosome, all other genes encoding components of the TNFα-related signalling pathway involved in the differentiation of skin appendages are located on the autosomes.

Pathogenic variants of the *WNT10A* gene (chromosome 2q35, OMIM 606268) are involved in the impairment of one or more ectodermal derivatives that cause HED, odonto-onycho-dermal dysplasia (OODD) (OMIM: # 257980; ORPHA: 2721), Schöpf-Schulz-Passarge syndrome (SSPS) (OMIM: # 224750; ORPHA: 50944) [9] and syndromic or non-syndromic NSTA (ORPHA: 99798) [10]. *EDA* pathogenic variants that underlie non-syndromic or syndromic oligodontia [11] have been described, likely because they alter a single signal transduction pathway. It has been postulated that X-linked hypohidrotic ectodermal dysplasia (XLHED) and EDA-related NSTA are the same disease with different degrees of expressivity [12].

In this study, we evaluated, for the first time, the mutational spectrum in the Spanish population with impairment of one or more ectodermal derivatives. We completely sequenced the *EDA*, *EDAR*, *EDARADD* and *WNT10A* genes in a large cohort of 72 unrelated patients.

## Materials and methods

### Subjects

A cohort of 72 Spanish subjects from a multicenter cross-sectional study with clinical signs of ectodermal derivative impairment was recruited; 63 (87.5%) cases were referred to as HED, and 9 (12.5%) were referred to as non-syndromic tooth agenesis (NSTA). Among these, 50 involved males and 22 involved females with ages ranging from 6 months to 69 years, most of whom were children (< 18 years) (84.7%). Clinical data and family history were collected with a specific questionnaire, and blood samples for genetic analysis were collected after written informed consent was obtained from patients or, in the case of minors, from their parents. The study was approved by the Virgen de la Arrixaca University Hospital Ethical Committee and adhered to the Helsinki Declaration.

### Sequencing analysis

Genomic DNA was extracted from peripheral blood lymphocytes using the commercial Maxwell® 16 blood DNA purification kit (Promega Corporation, Madison, WI, USA) with the automatized system Maxwell® 16 (Promega Corporation, Madison, WI, USA). Primers covering the exons and a minimum of 20 bp in the flanking intronic sequences of the *EDA*, *EDAR*, *EDARADD* and *WNT10A* genes were designed with Prime3Plus [13]. PCR was performed, and the products were sequenced with an ABI3100 genetic analyser (Applied Biosystems, Foster City, CA, USA). The electropherograms were analysed with Sequence Scape Software v3.0 (Applied Biosystems, Foster City, CA, USA). A mutation nomenclature was used in which + 1 corresponded to the A of the ATG translation initiation codon of the reference sequence NM_001399.4 (*EDA*), NM_022336.3 (*EDAR*), NM_145861.2 (*EDARADD*) or NM_025216.2 (*WNT10A*). All detected variants were assessed using the Mutation Taster [14], SIFT [15], PolyPhen2 [16] and PROVEAN [17] in silico human genome variant prediction tools. The interpretation of the sequence variants was performed according to the American College of Medical Genetics and Genomics (ACMG) guidelines [18]. This study did not include causative mutations in remote intronic or regulatory regions of genes.

### Assessment of copy number variation by MLPA

Multiple ligation-dependent probe amplification (MLPA) was performed with the SALSA MLPA P183-C1 EDA-EDAR-EDARAD probe mix commercial kit and the SALSA MLPA EK1 Cy5 reagent kit (MRC-Holland, Amsterdam, Netherlands) according to the manufacturer’s instructions. The fragment sizes were determined by capillary electrophoresis using the above-mentioned nucleic acid analyser. The relative exon copy numbers were calculated after normalization of the peak height value of the patients against the mean of the peak height value of the control group (at least two individuals of the same sex were known to have normal gene dosage) using the Coffalyser. Net software by MRC-Holland.

### Pattern of X inactivation

The X inactivation patterns were analysed using an assay of the polymorphic CAG repeat in exon 1 of the androgen receptor gene (AR). The digestion of peripheral blood DNA with the methylation-sensitive restriction enzyme HpaII resulted in cleavage only in restriction sites of active X-chromosomes, while the inactive X-chromosomes remained intact. The direction of skewing was determined in female carriers when the parental mutation origin was confirmed by segregation analysis of the polymorphic repeat in AR. An X-chromosome inactivation pattern that was skewed 80:20 or less was classified as random, while a pattern skewed between 80:20 and 90:10 was classified as moderately skewed, and a pattern skewed more than 90:10 was considered highly skewed [19].

## Results

The average age at genetic diagnosis was 5.4 years in children (42/51) and 40.2 years in adult patients (9/51). Pathogenic variants were identified in coding sequences or flanking intronic regions in 70.8% (51/72) of patients. Among the patients, 72.5% (37/51) had variants of *EDA*, 15.7% (8/51) had variants of the *WNT10A* gene, 7.8% (4/51) had variants of EDAR and 9.8% (5/51) had variants of *EDARADD*. In most cases, the variants were inherited (82.4%; 42/51), and the variants were inherited according to an X-linked (64.3%; 27/42), autosomal dominant (23.8%; 10/42) or autosomal recessive pattern (11.9%; 5/42). Regarding the phenotype, pathogenic variants were identified in 74.6% (47/63) of HED patients and 44.4% (4/9) of NSTA patients. *EDA* was the most frequently involved gene in HED patients (76.6%; 36/47). In NSTA patients, *WNT10A* variants were present in 3 cases, and an *EDA* variant was present in one case. A total of 46 different pathogenic variants (76.6%) were identified among 60 variants in 51 patients. The allelic heterogeneity was 86.4% (32/37) for *EDA*, 100% (5/5) for *EDAR,* 20% (1/5) for *EDARADD* and 66.6% (8/13) for *WNT10A.* The observed variant types were complete EDA gene deletion [1], EDA exon 1 deletion [1], missense variants [20], nonsense variants [7] small indels [4], frameshift variants [6] and intronic variants [2]. Most pathogenic variants were located in conserved and functionally relevant domains in known hotspots. Twelve out of 46 variants (26.0%) were novel and had not been previously reported among the 471 variant entries for these four genes in the Human Gene Mutation Database (HGMD®) [21] (*last consultation on August, 15, 2019)*.

*EDA* was the most frequently involved gene (72.5%), as previously mentioned, with pathogenic variants in 30 males and 7 females. Of these, 32 variants were different, and 9 were novel and more frequently located in exons 1, 2, 4, 7 and 8. Seventy-five percent of variants were inherited (27/36) and most were found in only one family, except for the complete exon 1 deletion (two cases), c.572_598del18/p.Pro191_Pro196del (three cases), c.467G > A/p.Arg156His in the furin domain (two cases) and c.1045G > A/p.Ala349Thr (two cases). Regarding the predicted functional impact of the variants, three of them resulted in the complete disruption of the protein, and the rest were distributed within coding regions in the transmembrane domain [2], furin cleavage site [4], collagen-like domain [10] and tumour necrosis factor homology domain [15] (Table 1).

**Table 1:** Allelic variants identified in the *EDA* gene

Family	Gender	NM	Exon/Intron	Protein	Affected domain	Variant	Origin	Clinical Diagnosis	HGMD (*)
10	F	Gene deletion	Complete	(−)	Gene loss	*Deletion*	De novo	HED	[22]
13	M	c.58C > T	Exon 1	p.Arg20Ter	Transmembrane domain	*Nonsense*	Inherited	HED	[23]
26	M	c.164 T > C	Exon 1	p.Leu55Pro	Transmembrane domain	*Missense*	Inherited	HED	Novel
20	M	Exon 1 deletion	Exon 1	(−)	Complete protein loss	*Deletion*	Inherited	HED	[24]
33	F	Exon 1 deletion	Exon 1	(−)	Complete protein loss	*Deletion*	De novo	HED	[24]
35	M	c.457C > T	Exon 2	p.Arg153Cys	Furin subdomain	*Missense*	De novo	HED	[25]
34	M	c.463C > T	Exon 2	p.Arg155Cys	Furin subdomain	*Missense*	De novo	HED	[26]
45	M	c.467G > A	Exon 2	p.Arg156His	Furin cleavage site	*Missense*	Inherited	HED	[26]
47	M	c.467G > A	Exon 2	p.Arg156His	Furin cleavage site	*Missense*	Inherited	HED	[26]
31	F	c.516delC	Exon 3	p.Asn172Lysfs*108	(−)	*Frame Shift*	De novo	HED	Novel
17	M	c.546_581del36	Exon 4	p.Asn185_Pro196del	Collagen-like domain	*In-Frame Deletion*	Inherited	HED	[26]
37	M	c.553_588del36	Exon 4	p.Asn185_Pro196del	Collagen-like domain	*In-Frame Deletion*	Inherited	HED	[27]
21	M	c.572_589del18	Exon 4	p.Pro191_Pro196del	Collagen-like domain	*In-Frame Deletion*	Inherited	HED	[28]
23	M	c.572_589del18	Exon 4	p.Pro191_Pro196del	Collagen-like domain	*In-Frame Deletion*	Inherited	HED	[28]
48	M	c.572_589del18	Exon 4	p.Pro191_Pro196del	Collagen-like domain	*In-Frame Deletion*	Inherited	HED	[28]
50	M	c.599C > G	Exon 4	p.Pro200Arg	Collagen-like domain	*Missense*	Inherited	HED	Novel
36	F	c.612delG	Exon 4	(−)	Collagen-like domain	*Frame Shift*	De novo	HED	Novel
02	M	c.612_629del18	Exon 4	p.Ile205_Gly210del	Collagen-like domain	*In-Frame Deletion*	Inherited	HED	[29]
06	M	c.653G > T	Exon 4	p.Gly218Val	Collagen-like domain	*Missense*	Inherited	HED	[30]
01	M	c.663_697del35	Exon 4	p.Pro222Thrfs*	Collagen-like domain	*Frame Shift*	Inherited	HED	[26]
29	M	c. 728_735del8	Exon 5	p.Thr243Lysfs*20	(−)	*Frame Shift*	De novo	HED	[24]
11	F	c.729_735del7	Exon 5	p.Arg244Thrfs*34	(−)	*Frame Shift*	Inherited	HED	Novel
14	M	c.760C > T	Exon 6	p.Gln254*	TNF homology domain	*Nonsense*	De novo	HED	Novel
22	M	c.793 + 1G > C	Intron 6	(−)	TNF homology domain	*Splicing*	Inherited	HED	[25]
51	M	c.866G > A	Exon7	p.Gly289His	TNF homology domain	*Missense*	ND	NSTA	[31]
15	F	c.871G > C	Exon 7	p.Gly291Arg	TNF homology domain	*Missense*	Inherited	HED	Novel [28](#)
16	F	c.880G > T	Exon 7	p.Glu294Ter	TNF homology domain	*Nonsense*	De novo	HED	Novel
07	M	c.892G > T	Exon 7	p.Asp298Tyr	TNF homology domain	*Missense*	Inherited	HED	[32]
08	M	c.895G > A	Exon 7	p.Gly299Ser	TNF homology domain	*Missense*	Inherited	HED	[26]
09	M	c.895G > C	Exon 7	p.Gly299Arg	TNF homology domain	*Missense*	Inherited	HED	[33]
03	M	c.995G > A	Exon 8	p.Cys332Tyr	TNF homology domain	*Missense*	Inherited	HED	[24]
30	M	c.1027 T > G	Exon 8	p.Tyr343Asp	TNF homology domain	*Missense*	Inherited	HED	Novel
19	M	c.1037G > A	Exon 8	p.Cys346Tyr	TNF homology domain	*Missense*	Inherited	HED	[7]
24	M	c.1045G > A	Exon 8	p.Ala349Thr	TNF homology domain	*Missense*	Inherited	HED	[26]
04	M	c.1045G > A	Exon 8	p.Ala349Thr	TNF homology domain	*Missense*	Inherited	HED	[26]
32	M	c.1049G > A	Exon 8	p.Gly350Asp	TNF homology domain	*Missense*	Inherited	HED	[34]
25	M	c.1069C > T	Exon 8	p.Arg357Trp	TNF homology domain	*Missense*	Inherited	HED	[29]

*F* female, *M* male, *TNF* tumour necrosis factor, *NM* nucleotide mutation, *HED* hypohidrotic ectodermal dysplasia, (*) Reference in HGMD database, (#) Novel variant in a nucleotide resulting in an equivalent amino acid, *NSTA* non-syndromic tooth agenesis, *ND* no data

Male patients with *EDA* pathogenic variants presented the classical XLHED phenotype. *EDA* female carriers also showed the HED phenotype; 6 out of 7 had predicted variants with severe functional consequences, including the disruption of the protein by frameshift mutations [3], partial or total *EDA* gene deletion [2] or a novel missense mutation affecting an amino acid (c.871G > C/p.Gly291Arg) described as crucial for proper protein folding [25]. X-chromosome inactivation analysis was informative in 6 out of 7 female carriers and revealed a random pattern favouring either chromosome (5/6) or moderately skewed X-chromosome inactivation (1/6). The HED phenotype in EDA female carriers was not correlated with the X-chromosome inactivation pattern (Table 2).

**Table 2:** X-chromosome inactivation pattern in *EDA* carrier females

Family	Case	N° (CAG) repeat in AR (bp)			% Chromosome X inactivation			Clinical Diagnosis
		Variant	A1	A2	A1	A2		
10	Proband	EDA gene deletion	270	270	NI	NI	*–*	HED
10	Monozygotic twin sister	EDA gene deletion	270	270	NI	NI	*–*	HED
11	Proband	c.729_738del7	286	290	74.4%	25.6%	*–*	HED
15	Proband	c.871G > C	274	277 (*)	60%	40% (*)	*Random*	HED
15	Mother	c.871G > C	277 (*)	280	61.6% (*)	38.4%	*Random*	Normal
16	Proband	c.880G > T	268	270	43.5%	56.5%	*Random*	HED
16	Mother	(−)	265	270	19.8%	80.2%	*Moderately skewed*	Normal
31	Proband	c.516delC	271	277	40%	60%	*Random*	HED
31	Mother	(−)	277	277	NI	NI	*–*	Normal
33	Proband	Deletion of exon 1	277	294	80.4%	19.6%	*Moderately skewed*	HED
33	Mother	(−)	277	283	36%	64%	*Random*	Normal
36	Proband	c.612delG	286	292	76.8%	23.3%	*Random*	HED
36	Mother	(−)	286	292	55.9%	44.1%	*Random*	Normal

(*) Allele carrier of c.871G > C variant, *HED* hypohidrotic ectodermal dysplasia, *NI* non-informative

*EDAR* variants were detected in 4 HED patients. One of these was a compound heterozygous mutation comprising a splicing variant upstream of the first intron (c.52-2A > G) and a missense variant in the TNFR (tumour necrosis factor receptor) domain (c.212G > A/p.Cys71Tyr) with an autosomal recessive inheritance pattern (published data) [3]; the other three variants, which were located in the death domain (DD), showed an autosomal dominant inheritance pattern (Table 3). All the *EDAR* pathogenic variants had been reported previously in the HGMD. HED patients with *EDAR* pathogenic variants were clinically indistinguishable from those patients with *EDA* variants. The Asian variant p.Val370Arg, which has been associated with an attenuated phenotype [35], was not observed in our cohort.

**Table 3:** Allelic variants identified in the *EDAR* gene

Family	Gender	NM	Exon/Intron	Protein	Affected domain	NM	Exon/Intron	Protein	Affected domain	Origin	Clinical Diagnosis	HGMD (*)
5	M	c.52-2A > G	Intron 2	(−)	Protein disruption	c.212G > A	Exon 4	p.Cys71Tyr	TNF Receptor	Inherited/Inherited	HED	[3]
18	M	c.1072C > T	Exon 9	p.Arg358*	Death domain	(−)	(−)	(−)	(−)	Inherited	HED	[26]
42	M	c.1073G > A	Exon 9	p.Arg358Gln	Death domain	(−)	(−)	(−)	(−)	Inherited	HED	[23]
12	F	c.1259G > A	Exon 12	p.Arg420Gln	Death domain	(−)	(−)	(−)	(−)	Inherited	HED	[26]

*F* female, *M* male, *HED* hypohidrotic ectodermal dysplasia, (*) Reference in HGMD database

In *EDARADD*, the c.308C > T/ p.Ser103Phe variant, which has been described as likely to be benign in ClinVar and of unknown significance in the HGMD, was identified in 5 patients and was inherited in all cases (Table 4). Three of the patients carried additional variants; two were located in the EDA gene (c.572_589 del18 (family 21)) and were associated with the classical HED phenotype, and the other (c.866G > A (family 51) was identified in a patient with NSTA. In one female (family 28), an additional c. 682 T > C variant in the *WNT10A* gene was linked to NSTA. In two HED patients, p.Ser103Phe in EDARADD was the only identified variant.

**Table 4:** Allelic variants identified in the *EDARADD* gene

Family	Gender	NM	Exon/Intron	Protein	Origin	Clinical Diagnosis	HGMD (*)	Second variant
21	M	c.308C > T	Exon 6	p.Ser103Phe	Inherited	HED	[36]	EDA: c.572_589del18
38	F	c.308C > T	Exon 6	p.Ser103Phe	Inherited	HED	[36]	
39	M	c.308C > T	Exon 6	p.Ser103Phe	Inherited	NSTA	[36]	WNT10A: c.682 T > A
40	M	c.308C > T	Exon 6	p.Ser103Phe	Inherited	HED	[36]	
51	M	c.308C > T	Exon 6	p.Ser103Phe	ND	NSTA	[36]	EDA: c.866G > A

*F* female, *M* male, *HED*, hypohidrotic ectodermal dysplasia, (*) Reference in HGMD database, *NSTA* non-syndromic tooth agenesis, *ND* no data

In the *WNT10A* gene, 8 different pathogenic variants were identified in 8 families, three of which were novel variants. A broader phenotypic spectrum was observed in this group of patients, and the most disruptive biallelic *WNT10A* mutations (in patients with homozygous or compound heterozygous mutations) were associated with Schöpf-Schulz-Passarge syndrome (variants c.18_43del26/p.Arg7Alafs*28, c.321C > A/p.Cys107* and c.1131C > A/p.Cys377*). Other significant variants have been found to be associated with HED (compound heterozygous c.27G > A/p.Trp9* and c.92 T > A/p.Leu31Glu) or normohidrotic ED (c.18_43del26/p.Arg7Alafs*28 and c.1131C > A/p.Cys377*). The recurrent *WNT0A* variant (c.18_43del26/p.Arg7Alafs*28) was found in two non-related patients from the same Spanish geographical region. One heterozygous patient was affected by odonto-onycho-dermal dysplasia (OODD) (c.514A > T/p.Arg172Trp) (Table 5).

**Table 5:** Allelic variants identified in the *WNT10A* gene

Family	Gender	NM	Exon/Intron	Protein	NM	Exon/Intron	Protein	Origin	Clinical Diagnosis	HGMD (*)
27	F	c.1A > T	Exon 1	p.M1?	c.321C > A	Exon 2	p.Cys107*	Inherited/Inherited	NSTA+	[37]/ [38]
46	F	c.27G > A	Exon 1	p.Trp9*	c.92 T > A	Exon 1	p.Leu31Glu	Inherited/Inherited	HED	[38]/Novel
43	F	c.18_43del26	Exon 1	p.Arg7Alafs*28	c.18_43del26	Exon 1	p.Arg7Alafs*28	ND	SSPS	Novel
44	F	c.18_43del26	Exon 1	p.Arg7Alafs*28	c.1131C > A	Exon 4	p.Cys377*	Inherited/Inherited	Normohidrotic ED	Novel/Novel
41	M	c.514A > T	Exon 2	p.Arg172Trp	(−)	(−)	(−)	Inherited	OODD	[39]
28	M	c. 682 T > A	Exon 3	p.Phe228Ile	(−)	(−)	(−)	Inherited	NSTA	[38]
39	M	c. 682 T > A	Exon 3	p.Phe228Ile	(−)	(−)	(−)	Inherited	NSTA	[38]
49	M	c.321C > A	Exon 2	p.Cys107*	c.321C > A	Exon 2	p.Cys107*	Inherited/Inherited	SSPS	[38]

*F* female, *M* male, *HED* hypohidrotic ectodermal dysplasia, (*) Reference in HGMD database, *NSTA* non-syndromic tooth agenesis, *NSTA+* non-syndromic tooth agenesis with other minor ectodermal anomalies, *SSPS* Schöpf-Schulz-Passarge, *ND* no data

In the NSTA group, a compound heterozygous patient from family 27 (c.1A > T/p.M1? and c.321C > A/p.Cys107* variants) showed oligodontia and subtle ectodermal-related symptoms, including thin hair with normal density, periorbital pigmentation and fragile nails. A milder phenotype was observed in two NSTA patients, both of whom had maxillary lateral incisor agenesis, carrying the c.682 T > A/ p.Phe228Ile heterozygous variant, the pathogenicity of which has been established based on the location of the mutation in an important functional domain of the protein; this protein has been implicated in interactions with the membrane receptor and, as consequence, in intra-cellular Wnt signalling [40].

## Discussion

The yield of the analysis of these four genes was 70.8%; specifically, the yield was 76.1% for HED and 44.4% for NSTA. Our cohort was similar in size to a French cohort described by Cluzeau et al. [7] with 61 HED patients; however, the four genes accounted for a larger proportion of the French cases (92%). This discrepancy may be due to recruitment and/or population differences.

*EDA* variants accounted for most cases (72.5%), followed by *WNT10A* (15.7%), *EDARADD* (9.8%) and *EDAR* (7.8%). The *WNT10A* gene should be considered the second candidate gene responsible for ectodermal derivative impairment, in accordance with recently published data for the Italian population [41].

There were more affected males (ratio 5:2) due to a higher prevalence of pathogenic variants in the *EDA* gene associated with XLHED. The mean age of genetic diagnosis was 5.4 years in children and 40.2 years in adult patients, which was ascertained through the genetic counselling process. The presence of adult patients clinically diagnosed after the age of forty without molecular characterization is significant, reflecting the pre-molecular stage. Taking into account that most detected variants were inherited, we should emphasize the importance of early genetic diagnosis and counselling to prevent new severe cases in affected families and to give these families the opportunity to utilize potential new genetically personalized therapies. Recently, a new prenatal treatment in patients with *EDA* variants [42] has been described that shows promise for most HED families.

Our cohort shows a higher allelic heterogeneity of 76.6% for the four analysed genes, 86.4% for *EDA* and 66.6% for *WNT10A*; in comparison, the highest published rates are 84% for *EDA* [27] and 42.4% for *WNT10A* [41].

In regard to the type and location of variants in the *EDA* gene, four in-frame deletions in the collagen-like domain in exon 4 have been identified (Table 1). Two of them, c.612_629del18 [29] and c.572_589del18 [28], have been previously described to be associated with hypomorphic phenotypes. The other two variants, c.546_581del36 (family 17) and c.553_588del36 (family 37), also result in less severe signs of HED and nearly normal sweating. In the patients with these variants, the in-frame deletion of the 19 Gly-X-Y repeats in the protein would produce a shorter collagen helix resulting from polymerase slippage but would not affect the multimerization and functionality, causing a milder phenotype [43].

Interestingly, all analysed *EDA* female carriers showed an HED phenotype that was not linked to skewed X-chromosome inactivation. It is important to note that X-chromosome inactivation was studied in peripheral blood cells instead of skin cells, which may show a different skewed pattern. However, on the other hand, the HED phenotype in these females may suggest that a biological mechanism other than X-inactivation is responsible, in addition to the possible influence of the specifically detected variants and other additional unknown genetic modifiers of clinical expressivity.

Regarding the *EDAR* variants (Table 3), two of them involve the evolutionally conserved residue Arg358 within the death domain (DD), which has been identified in patients from different continents (c.1072C > T/p.Arg358Ter in American [44] and c.1073G > A/p.Arg358Gln in Asiatic families) [23]. The missense mutation in Arg358 may not affect the interaction with *EDARADD* [45].

The only identified variant in *EDARADD* (p.Ser103Phe), which was found in 5 patients, is quite prevalent in the European population. Although the allelic frequency in the healthy population of this variant was 2% according to the dbSNP database, some authors have suggested that it may make a significant contribution to NSTA but show a low penetrance [29]. In addition, it has been associated with a more severe phenotype in combination with other variants in a heterozygous state in a recent publication [46]. The phenotypes of our patients with p.Ser103Phe variant were consistent with both HED (4/5) and NSTA (1/5) (Table 4). The clinical features in three of them can be explained by the presence of an additional pathogenic variant in *EDA* and *WNT10A*. However, in the other two patients with the classical HED phenotype, unknown additional variants in other genes are expected to be involved.

In terms of phenotypes, the *EDA* gene was most frequently involved in HED (76.6%) patients, followed by both *EDAR* and *EDARADD* and, to a lesser extent, WNT10A. In the Cluzeau cohort [6], the *EDA* gene accounted for a lower proportion of HED cases (58%). Our results support the choice to study the *EDA* gene first, due to its significantly high yield, without using NGS technology in a Spanish patient showing the classical triad of HED symptoms.

The *WNT10A* gene has been associated with a wide spectrum of ectodermal derivative impairment manifestations, ranging from NSTA to complex rare syndromes such as OODD and SSPS [9, 10, 29, 37, 38, 41, 47–49]. Our findings (Table 5) also reveal that heterozygous variants in WNT10A are associated with NSTA, while homozygous or compound heterozygous variants are linked to a more severe phenotype, either OODD and SSPS, as previously described. Recently, WNT10A-linked oligo/hypodontia phenotypes have been reported to be associated with minor ectodermal manifestations, such as mild hair, nails and sweating anomalies [6], as described in our patient from family 27 with tooth agenesis and minor ectodermal signs (NSTA+). For this reason, it is important to take *WNT10A* into account as a candidate gene for clinical conditions characterized by dental agenesis and other minor ectodermal features, especially in the absence of typical HED facial dysmorphism.

Furthermore, we found that a proportion of cases of tooth agenesis [50], orodental involvement [20] or skin disease [51] may be explained by polygenic inheritance with the co-segregation of multiple variants, which may modulate the final phenotype [52], emphasizing the need to apply more powerful molecular analysis tools during ED diagnosis [39]. In the near future, we will need to understand the pathogenesis and impact of the combination of different allelic variants in different genes in addition to those involved in the Eda or Wnt signalling pathways, mainly by considering that these pathways are associated with specific adaptations in the natural population and that some variants attenuate or increase the final effects; this reflects the relationship between human disease and natural variation, as has been hypothesized [53].

## Conclusions

This is the only molecular study conducted to date in the Spanish ED population, resulting in the specific genetic diagnosis of affected families with HED and NSTA. *EDA*, *EDAR*, *EDARADD* and *WNT10A* genes constitute the molecular basis of disease in 70.8% of the patients, with a 74.6% yield for HED and 44.4% for NSTA. A high allelic heterogeneity was revealed mainly in *EDA, EDAR* and *WNT10A*, for which 12 novel variants were identified. *EDA* is the most prevalent gene in our cohort, which supports the study of the *EDA* gene first, due to its significantly high yield, in Spanish patients showing the classical triad of HED symptoms who do not have access to NGS technology. Our data also confirm that the *WNT10A* gene is the second molecular candidate for involvement in ectodermal derivative impairment, accounting for one-half of non-*EDA* patients and one-third of NSTA patients.

The broad phenotype spectrum (spanning from classical HED to NSTA) points to the need for a multidisciplinary approach for the care of these patients. The early recognition of these phenotypes and molecular genetic diagnosis in childhood are essential to provide accurate genetic counselling and access to potential new treatments. Further studies using NGS will help to identify the other genes involved in the remaining uncharacterized Spanish patients.

## Data Availability

The datasets used and/or analysed during the current study are available from the corresponding author upon reasonable request.
